# FBXO44-Mediated Degradation of RGS2 Protein Uniquely Depends on a Cullin 4B/DDB1 Complex

**DOI:** 10.1371/journal.pone.0123581

**Published:** 2015-05-13

**Authors:** Benita Sjögren, Steven Swaney, Richard R. Neubig

**Affiliations:** 1 Department of Pharmacology & Toxicology, Michigan State University, 1355 Bogue Street, East Lansing, MI 48824, United States of America; 2 Center for Chemical Genomics, University of Michigan, 210 Washtenaw Avenue, Ann Arbor, MI 48109, United States of America; University of North Dakota, UNITED STATES

## Abstract

The ubiquitin-proteasome system for protein degradation plays a major role in regulating cell function and many signaling proteins are tightly controlled by this mechanism. Among these, Regulator of G Protein Signaling 2 (RGS2) is a target for rapid proteasomal degradation, however, the specific enzymes involved are not known. Using a genomic siRNA screening approach, we identified a novel E3 ligase complex containing cullin 4B (CUL4B), DNA damage binding protein 1 (DDB1) and F-box protein 44 (FBXO44) that mediates RGS2 protein degradation. While the more typical F-box partners CUL1 and Skp1 can bind FBXO44, that E3 ligase complex does not bind RGS2 and is not involved in RGS2 degradation. These observations define an unexpected DDB1/CUL4B-containing FBXO44 E3 ligase complex. Pharmacological targeting of this mechanism provides a novel therapeutic approach to hypertension, anxiety, and other diseases associated with RGS2 dysregulation.

## Introduction

The ubiquitin-proteasomal pathway of protein degradation controls critical biological functions including cell cycle and gene transcription [1–4] and dysregulation can result in accumulation of misfolded proteins, cell cycle arrest and uncontrolled cell proliferation. Consequently, disease states such as cancer and cardiovascular disease can be related to defects in this machinery [1,5–8]. This intricate system involves the coupling of a chain of ubiquitin molecules onto the target protein through a series of enzymes; E1, ubiquitin activating enzyme; E2, ubiquitin conjugating enzyme and E3 ligases. The ubiquitin chain is then recognized by the 26S proteasome, which degrades the target protein. The diverse and complex mechanisms for proteasome substrate recognition [4] arises from the large family (>600) of mammalian E3 ligases [2].

General proteasome inhibitors, such as Bortezomib (PS-341; Velcade) and carfilzomib have found value for the treatment of multiple myeloma and other cancers [9,10]. Not surprisingly, given the numerous processes regulated by the proteasome, these drugs are associated with a broad array of side effects. More selective strategies such as targeting specific E3 ligases have recently been successful in cancer drug discovery with the development of several inhibitors of the tumor suppressor p53 binding to its E3 ligase MDM2 [11–15]. However, further insight into specific E3 ligase selectivity is needed to apply this strategy to other clinically relevant degradation pathways.

Regulator of G Protein Signaling (RGS) proteins have received increasing attention as drug targets [16–20]. RGS proteins reduce the amplitude and duration of signaling through G protein-coupled receptors (GPCRs) through their GTPase accelerating protein (GAP) activity towards active (GTP-bound) Gα subunits of heterotrimeric G proteins [20,21]. Many clinically used drugs (~25–40%) act on GPCRs or related processes so there is a huge potential for RGS proteins in drug discovery. In the past decade several RGS inhibitors have been described [22–24], however, increasing the activity of a protein using small molecules is challenging.

RGS2 is widely expressed throughout the cardiovascular system (e.g. heart, kidney and vascular smooth muscle) as well as in the central nervous system [25–29]. It inhibits signaling through a number of GPCRs mediating vasoconstriction, such as Angiotensin II and Endothelin-1 receptors and consequently RGS2^-/-^ mice exhibit hypertension and prolonged responses to vasoconstrictor agents [30]. Furthermore, decreased protein levels (and activity) of RGS2 have been implicated in the progression of prostate cancer [31] and anxiety [32–34]. Thus, finding selective ways to increase RGS2 protein levels could have broad clinical implications. We previously showed that digoxin-mediated stabilization of RGS2 protein levels has functional effects on GPCR signaling [35], demonstrating that increased RGS2 protein levels correlates with enhanced functionality.

RGS2 has a very short protein half-life due to rapid proteasomal degradation [35,36] and general proteasome inhibitors, such as MG-132, significantly increase RGS2 protein levels *in vitro* [35]. For the closely related RGS4 and RGS5 proteins the precise molecular mechanism for protein degradation has been described [37–39]. However, the enzymes that are responsible for RGS2 protein degradation have yet to be identified. The elucidation of these mechanisms would provide novel, selective strategies for the development of small-molecule stabilizers of RGS2.

In the current study we used high-throughput siRNA screening to identify genes that are involved in RGS2 protein degradation. Hits, or those genes that, when removed, increased RGS2 protein levels were confirmed by siRNA knock-down and overexpression studies as well as effects on RGS2 protein half-life. We further demonstrated association between RGS2 and degradation components by a series of co-immunoprecipitation studies. Together these experiments led to the identification of a novel cullin 4B (CUL4B)/DNA damage binding protein (DDB1)/F-box 44 (FBXO44) E3 ligase complex responsible for RGS2 protein degradation. We also identify the first association of an F-box-only protein with a CUL4 complex. Although FBXO44 was also found to associate with its cognate partners Skp1 and CUL1, that complex is unable to degrade RGS2, highlighting the complexity of substrate recognition mechanisms within the ubiquitin-proteasomal pathway. These new findings reveal possible novel drug targets for selective enhancement of RGS2 function.

## Experimental Procedures

### Materials

MG-132 was purchased from Calbiochem (Quincy, MA). If not otherwise indicated all chemicals were from Sigma-Aldrich (St Louis, MO) and all tissue culture supplies were from Invitrogen/Gibco (Grand Island, NY). siGENOME SMART-POOL siRNA was obtained from Dharmacon/GE Healthcare (Lafayette, CO)

### DNA constructs

pcDNA3.1-RGS2-HA and pcDNA3.1-RGS4-HA have been previously described [36] as have pCMV-C3-RGS2-ProLabel and pCMV-C3-RGS4-ProLabel [35]. FLAG-FBXO44 and FLAG-FBXO44ΔN were gifts from Kevin Glenn, University of Iowa. pcDNA3-myc3-CUL4A and pcDNA3-myc3-CUL4B were obtained through Addgene [40].

### Antibodies

Rat anti-HA was from Roche (11867423001; Pleasanton, CA) and rabbit anti-ubiquitin FK1 was from Millipore (Billerica, MA). Mouse anti-FLAG (F3040), rabbit anti-FLAG (F7425), rabbit anti-CUL4A (SAB1411512), rabbit anti-CUL4B (SAB2107673), rabbit anti-FBXO44 (HPA003363), Rabbit anti-rat-HRP (A5795) and goat anti-rabbit-HRP (A0545) were from Sigma-Aldrich. Rabbit anti-CUL1 (#4995), rabbit anti-Skp1 (#12248) and rabbit anti-DDB1 (#6998) were from Cell Signaling (Danvers, MA). Rabbit anti-HA (sc-805), goat anti-Actin-HRP (sc-1615) and goat anti-mouse-HRP antibody (sc-2060) were from Santa Cruz (Santa Cruz, CA). Rabbit anti-RGS2 antibody was a gift from Dr. David Siderovski, University of North Carolina.

### Cells and transfections

The culture conditions of HEK-293 cells and the development of stable HEK-293 cell lines expressing RGS2-ProLabel or RGS4-ProLabel has been previously described [35]. Cells were transfected with DNA plasmids using Lipofectamine 2000 (Invitrogen) under serum free conditions in Opti-MEM (GIBCO, 31985). For transient transfections, experiments were run 24 h after transfection.

siRNA transfections were performed using 25 nM siGENOME SMART-POOL siRNA (Dharmacon/Thermo Scientific, Pittsburgh, PA) using Lipofectamine 2000; experiments were performed 48 h after transfection.

### Celltiter Fluor viability and PathHunter ProLabel assay

The multiplex procedure for the Celltiter Fluor viability assay and the PathHunter ProLabel assay has been previously described [35]. The PathHunter ProLabel protein expression assay (DiscoveRx) was performed immediately following the viability assay. Chemiluminescence corresponding to relative RGS2 or RGS4 protein expression was detected on a Pherastar plate reader (BMG, Cary, NC).

### siRNA screen

The druggable genome subset of the human siGENOME SMART-POOL siRNA library [41] was introduced into RGS2-PL cells through reverse transfection in 384-well plates (Corning #3570, Tewksbury, MA). Briefly, 5 μl siRNA (250 nM; final concentration 25 nM) was spotted in each well. Non-targeting siRNA (SMART-POOL #1) was used as background control and siTOX, PLK1 and Kif-11 were used as transfection efficiency controls. Lipofectamine RNAiMAX was diluted in OPTI-MEM to 40 μl/ml and incubated at room temperature for 10 min. 5 μl (0.2 μl RNAiMAX/well) was added to each well and the plates were incubated for 30 min at room temperature. Cells were trypsinized, counted in a Countess automatic cell counter (Invitrogen) and re-suspended in OPTI-MEM, 1% FBS to a concentration of 250,000 cells/ml. 40 μl of cell mixture was added to each well (10,000 cells/well). After 24 h, 5 μl Passive Lysis buffer (Promega, Madison, WI) was added in each corner well to create zero controls for the viability assay and 10 μM MG-132 was added as a positive control for RGS2 protein upregulation.

The Celltiter Fluor (Promega) and PathHunter ProLabel (DiscoveRx, Freemont, CA) assays were performed 48h after transfection [35]. Data was uploaded to the MScreen database (Center for Chemical Genomics, University of Michigan) and analyzed for genes that, when knocked down, increased RGS2-PL levels >3 SD above levels with non-targeting siRNA.

### Bioinformatic analysis

To assess global gene expression levels in HEK 293 cells without siRNA knockdown http://www.ncbi.nlm.nih.gov/geo/query/acc.cgi?acc=GSM258517 "HEK293, Control RNA treatment" was chosen as characteristic of control expression levels. Each gene on this array was annotated according to the quantile associated with the observed expression level and these data were used to annotate genes found to be responsive to siRNA knockdown. GeneGo analysis with the "Analyze Network" algorithm was performed, using the siRNA responsive genes to seed the network. In addition, ConceptGen analysis of the siRNA response genes was performed to reveal enriched GO Molecular Function concepts.

### siRNA confirmation of degradation hits

Based on the bioinformatics analysis 21 genes involved in protein degradation were identified as hits in the siRNA screen for genes that regulate RGS2 protein levels. Each siGENOME SMART-POOL contains a mix of four siRNA oligos targeting different regions of the gene of interest. To confirm the results from the screen each individual oligo from the 21 protein degradation genes was ordered and subjected to the same assay as the primary screen. siRNA was introduced in the RGS2-PL cells through reverse transfection as described above and the Celltiter Fluor and PathHunter ProLabel assay was run 48h after transfection. For a gene to be considered a confirmed hit at least 2 of the 4 oligos needed to have an effect on RGS2 protein levels.

### Preparation of cell lysates

Cells were harvested and lysed on ice in RIPA buffer containing phosphatase and protease inhibitors (20 mM Tris-HCl, pH7.4, 150 mM NaCl, 1 mM EDTA, 1 mM β-glycerophospate, 1% Triton X-100, 0.1% SDS, 2mM Na_3_VO_4_, Complete protease inhibitor cocktail EDTA-free (Roche)). Lysates were transferred to plastic centrifuge tubes and sonicated for 10 min at 4°C, centrifuged at 500×*g* for 3 min and the supernatant used for SDS-PAGE and immunoblotting.

### Preparation of tissue lysates

Heart tissue from C57/Bl6 mice was homogenized using a bullet blender (NextAdvance) in 300 μl lysis buffer containing protease and phosphatase inhibitors (20 mM Tris-HCl (pH 7.4), 150 mM NaCl, 2 mM EDTA, 5% glycerol, 1% Triton-X100, 2 mM Na_2_VO_4_, Complete protease inhibitors, EDTA-free (Roche)).

### SDS-PAGE and Western blot

Protein concentration in the cell and tissue lysates was adjusted with Laemmli buffer (BioRad, Hercules, CA) and equal amounts of protein in each lane were resolved on a 12% SDS-PAGE gel for 1 h at 160 V. Samples were transferred to an Immobilon-P membrane (Millipore) and subjected to Western immunoblot analysis using Tris-buffered saline (10 mM Tris, pH 8.0, 150 mM NaCl), 0.1% Tween-20 (TBS-T), 5% (w/v) nonfat dry milk for blocking and antibody diluent. The membrane was washed four times after each antibody in TBS-T and the protein bands were visualized on autoradiography film using the Super Signal West Pico chemiluminescent substrate (Pierce).

### Co-immunoprecipitation

Cells were washed with ice-cold PBS and harvested in lysis buffer (20 mM Tris-HCl (pH 7.4), 150 mM NaCl, 2 mM EDTA, 5% glycerol, 1% Triton-X100, 2 mM Na_2_VO_4_, Complete protease inhibitors, EDTA-free (Roche)). Lysis proceeded on ice for 30 min followed by centrifugation for 10 min at 10,000×*g* followed by pre-clearing with 50 μl Protein G-agarose beads (Roche) and rotation for 30 min at 4°C. Tissue lysates were subjected to the same pre-clearing step. 1 mg/sample was subjected to immunoprecipitation using 1 μg of indicated antibodies and 50μl Protein G-agarose with constant rotation for 2 h at 4°C. Following several washes with lysis buffer, bound proteins were eluted in Laemmli buffer (BioRad, Hercules, CA) and subjected to SDS-PAGE and Western blot. In cases where only one antibody was available for a protein (such as for endogenous FBXO44) Protein A-HRP (Life Technologies) was used for detection.

### Data analysis

Western blot images were scanned and quantified using the ImageJ software (NIH, Bethesda, MD). All data were analyzed using GraphPad Prism 6.0 (GraphPad; La Jolla, CA). Dose response curves were fit using non-linear regression. Datasets were analyzed with one-way ANOVA with Bonferroni’s post hoc test for multiple comparisons. Data are presented as mean ± S.D. with a *p*-value less than 0.05 considered significant.

## Results

### Distinct mechanisms of RGS2 and RGS4 protein degradation

**Fig 1:**
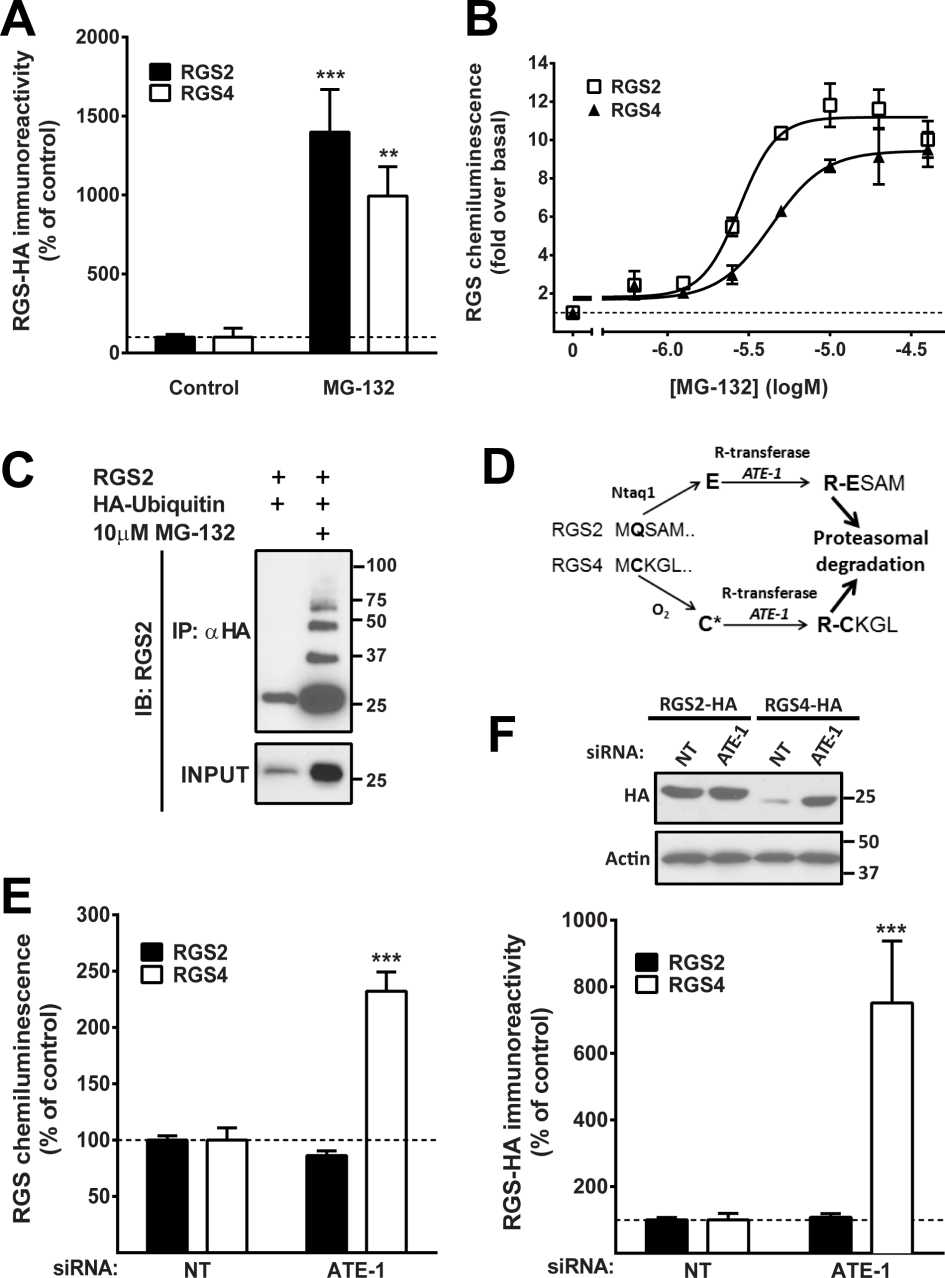
Protein expression of both RGS2 and RGS4 is dramatically increased by the proteasome inhibitor MG-132 as shown by Western blot (**A**) and the chemiluminescence-based PathHunter ProLabel assay (**B**). **C**. Co-immunoprecipitation of HEK-293 cells transiently transfected with RGS2 and HA-ubiquitin with or without treatment with 10μM MG-132. Proteasome inhibition results in a marked increase in protein levels and poly-ubiquitination of RGS2. **D**. The N-end rule pathway for the known substrate RGS4 and the hypothetical pathway for RGS2 (C; cysteine, G; glutamine; E; glutamic acid, R; arginine). *ATE-1* siRNA results in a significant increase in protein levels of RGS4, but has no effect on RGS2 protein levels by the PathHunter ProLabel assay (**E**) and Western blot (**F**). In both assays the protein levels of each RGS protein is compared to the levels with non-targeting siRNA (NT), but note that basal protein levels of RGS4-HA are much lower than RGS2-HA. Data are presented as mean ± S.D. of 3 independent experiments run in triplicate; **P<0.01; ***P<0.001 using one-way ANOVA with Bonferroni’s post hoc test for pairwise comparisons.

Both RGS2 and RGS4 are rapidly degraded by the proteasome [35,36]. In line with this, both RGS2 and 4 protein levels are increased [35,36], to a similar magnitude, by proteasome inhibition (Fig 1A and 1B). Furthermore, treatment with the proteasome inhibitor MG-132 results in poly-ubiquitination of RGS2 (Fig 1C and [42]), strongly implicating the ubiquitin-proteasomal pathway in RGS2 protein degradation.

RGS4 is a known substrate for the N-end rule pathway of proteasomal degradation and is targeted to this system by arginyl transferase (encoded by *ATE-1*) [37,38]. Based on its N-terminal sequence we had proposed [43] that RGS2 would also be an *ATE-1* substrate (Fig 1D). As expected [37,44], siRNA-mediated knock-down of *ATE-1* resulted in a substantial increase in RGS4 protein levels (Fig 1E and 1F). In contrast, RGS2 protein levels were unaffected by *ATE-1* siRNA. Consequently, RGS2 proteasomal degradation must utilize a different mechanism.

### High-throughput siRNA screen to identify genes involved in RGS2 protein degradation

We utilized the “druggable genome” subset of the Dharmacon siGENOME SMART-POOL siRNA library in a high throughput screen of changes in RGS2 protein levels. This library includes ~3,200 genes representing druggable targets, as well as a majority of genes (for proteases and Ub ligases) involved in protein degradation. As a robust platform to efficiently detect RGS2 protein levels we used the DiscoveRx PathHunter ProLabel HTS-compatible β-galactosidase complementation assay previously described [35] (Z-factor 0.72). The screen was performed in triplicate of each siRNA SMART-POOL in 384-well plates and cells were assayed for RGS2 expression 48 hours after transfection.

Hits were defined when RGS2 protein expression increased >3 S.D. above non-targeting siRNA for at least two out of the three siRNA replicates. Bioinformatic analysis and gene classifications of the hits are presented in S1 Table. Since the primary objective was to identify RGS2 degradation mechanisms we focused on genes involved in proteasomal degradation. Since the SMART-POOL siRNA contains four oligos, we used the individual oligos for follow-up, which revealed cullin 4B (CUL4B) and F-box 44 (FBXO44) as the top hits (S2 Table) so we investigated the role of these two proteins in RGS2 protein degradation.

### RGS2 protein levels are regulated by FBXO44 and CUL4B

**Fig 2:**
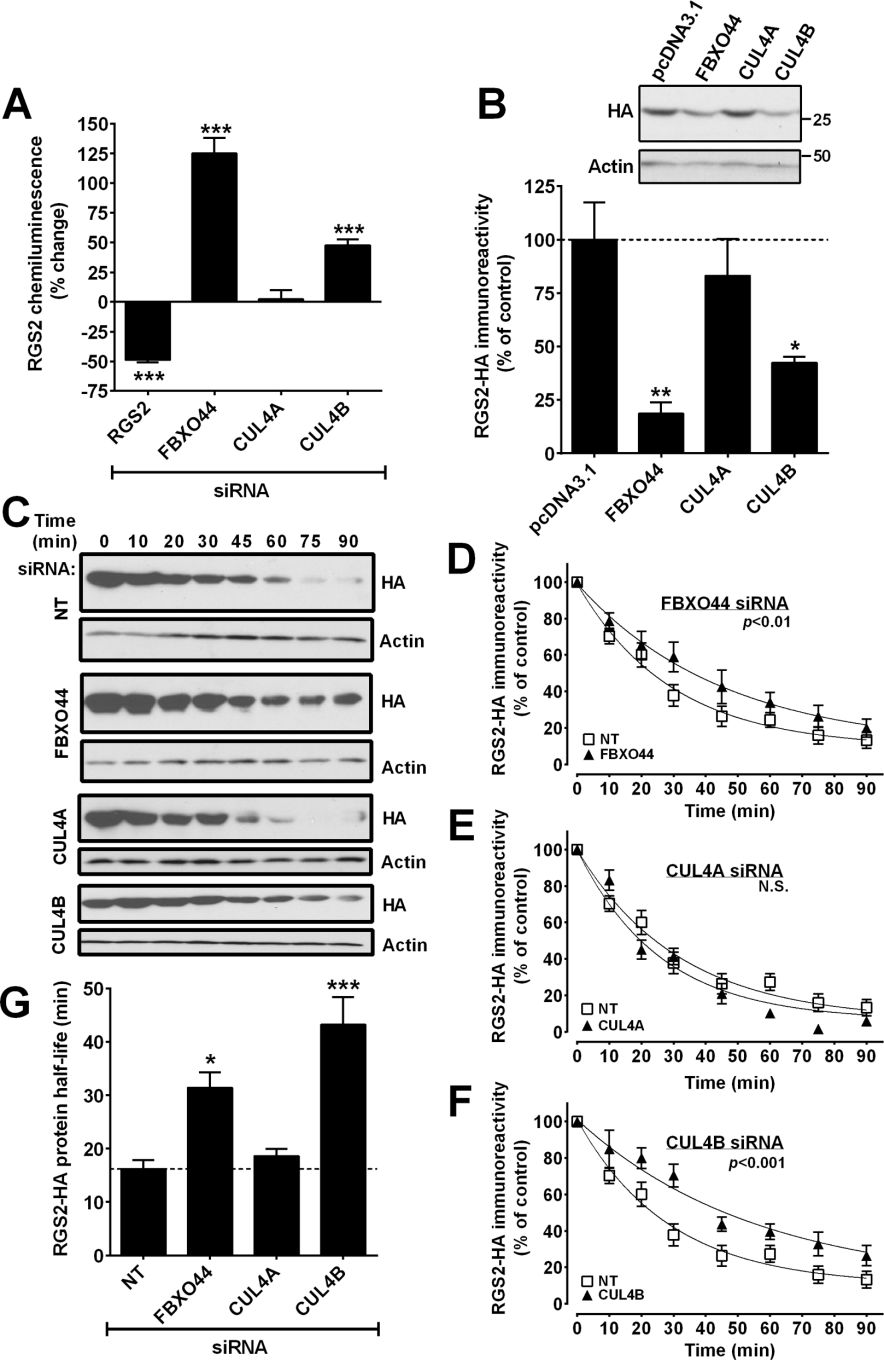
**A**. Increased RGS2 protein levels (PathHunter ProLabel assay) result from siRNA-mediated knockdown of CUL4B or FBXO44, but not of CUL4A **B**. Overexpression of CUL4B and FBXO44 decreases RGS2 protein levels whereas CUL4A overexpression has no effect. **C-G.** FBXO44 and CUL4B regulate RGS2 protein stability. HEK-293 cells were transiently transfected with RGS2-HA and treated with cycloheximide (10μg/ml) to inhibit protein synthesis. Representative Western blots (**C**) and time-course plots showing prolonged RGS2 protein half-life after either FBXO44 (**D**) or CUL4B (**F**) but not CUL4A (**E**) siRNA. Values were normalized to the level at 0 min (no cycloheximide), using actin as a loading control. **G.** Quantification of RGS2 protein half-life after siRNA-mediated knockdown of FBXO44, CUL4A and CUL4B. Data are presented as mean ± S.D. of 3 independent experiments run in triplicate; *P<0.05; **P<0.01; ***P<0.001 using one-way ANOVA with Bonferroni’s post hoc test for pairwise comparisons.

SiRNA-mediated knock-down of FBXO44 or CUL4B, but not CUL4A, resulted in a significant increase in RGS2 protein, in the PathHunter ProLabel assay (Fig 2A). Little is known about the function of FBXO44 and only a few specific functions have been attributed to CUL4B [45,46]. These results raise the possibility of a novel E3 ligase complex containing these two proteins in RGS2 degradation. Consistent with this, overexpression of either CUL4B or FLAG-FBXO44 significantly decreased RGS2 protein levels. In contrast, overexpression of CUL4A had no significant effect on RGS2 protein levels (Fig 2B).

If CUL4B and FBXO44 are involved in RGS2 degradation, then reducing their expression should prolong the half-life of RGS2 protein. To confirm this prediction, HEK-293 cells were transiently transfected with RGS2-HA and siRNA against FBXO44, CUL4A or CUL4B then treated with cycloheximide to inhibit protein synthesis. Consistent with our previous work [35], RGS2 had a very short protein half-life of 16±4 min with non-targeting siRNA (Fig 2C–2G) but was significantly increased when cells were transfected with siRNA against either FBXO44 (Fig 2C, 2D and 2G; t_1/2_ = 31±6 min) or CUL4B (Fig 2C, 2F and 2G; t_1/2_ = 43±13 min). CUL4A siRNA had no significant effect on RGS2 protein half-life (Fig 2C, 2E and 2G; t_1/2_ = 19±2 min), further establishing the selectivity between CUL4A and CUL4B in regulating RGS2 protein stability.

### FBXO44 associates with RGS2

**Fig 3:**
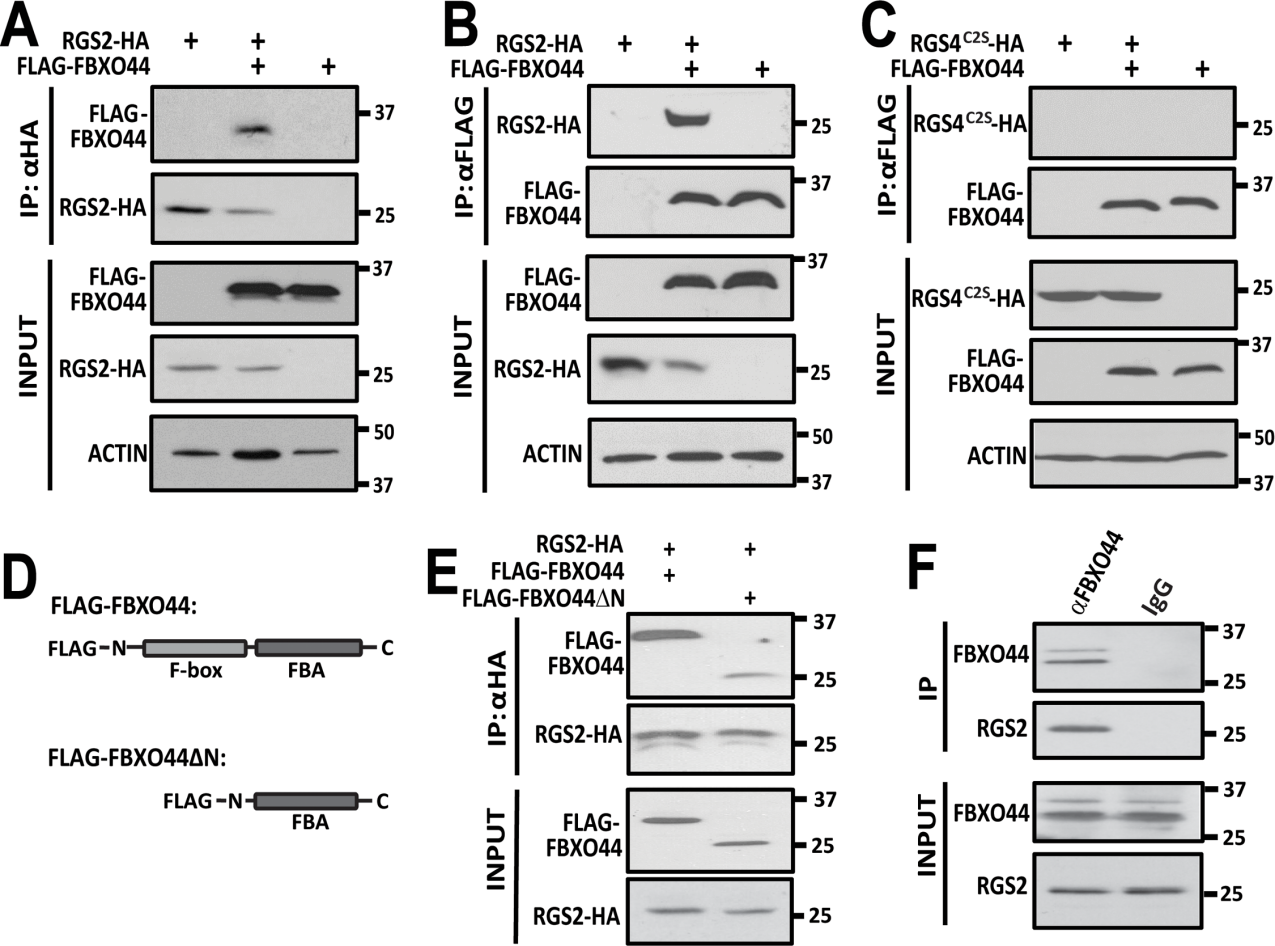
**A**. FLAG-FBXO44 co-immunoprecipitates with RGS2-HA. **B**. The reciprocal experiment shows that RGS2-HA co-immunoprecipitates with FLAG-FBXO44. Also note that co-expression with FLAG-FBXO44 reduces total protein levels of RGS2-HA. **C.** RGS4^C2S^-HA does not co-immunoprecipitate FLAG-FBXO44. The RGS4^C2S^-HA mutant was used due to the low protein expression obtained with wild-type RGS4-HA. **D.** Schematic of full length FBXO44 and FBXO44ΔN. **E.** RGS2-HA co-immunoprecipitates both full length FBXO44 and FBXO44ΔN. **F.** RGS2 immunoprecipitates with FBXO44 in lysates from mouse whole heart tissue.

Since F-box proteins are responsible for substrate recognition in cullin-RING ligase (CRL) complexes [47] one would expect FBXO44 to bind RGS2. Indeed, RGS2-HA co-immunoprecipitated FLAG-FBXO44 (Fig 3A). In the reciprocal experiment FLAG-FBXO44 could co-immunoprecipitate RGS2-HA (Fig 3B), suggesting that these two proteins can exist in a complex in cells. Reduction in RGS2 protein levels was again observed in both cases when FLAG-FBXO44 was co-expressed. In contrast, neither wild-type RGS4 (data not shown) nor a stabilized mutant of RGS4 (RGS4^C2S^; Fig 3C) immunoprecipitated with FLAG-FBXO44, further establishing the specificity of the FBXO44 mechanism for RGS2 over another closely related and rapidly degraded RGS protein.

FBXO44 belongs to the FBA (for F-box associated) family of F-box proteins that contain no distinct additional protein domains apart from the F-box domain, [48]. To determine the role of the F-box domain for association of FBXO44 with RGS2 we used FBXO44 lacking the N-terminus and F-box, FBXO44ΔN (Fig 3D). RGS2 could still co-immunoprecipitate FBXO44ΔN (Fig 3E), showing that the N-terminus and F-box domain are not essential for association with RGS2.

To further validate the association between RGS2 and FBXO44 we demonstrated successful co-immunoprecipitation of endogenous proteins from mouse heart tissue lysate (Fig 3F). These data confirm the association between RGS2 and FBXO44 in a biologically relevant system.

### CUL4B, but not CUL1, regulates RGS2 protein levels

**Fig 4:**
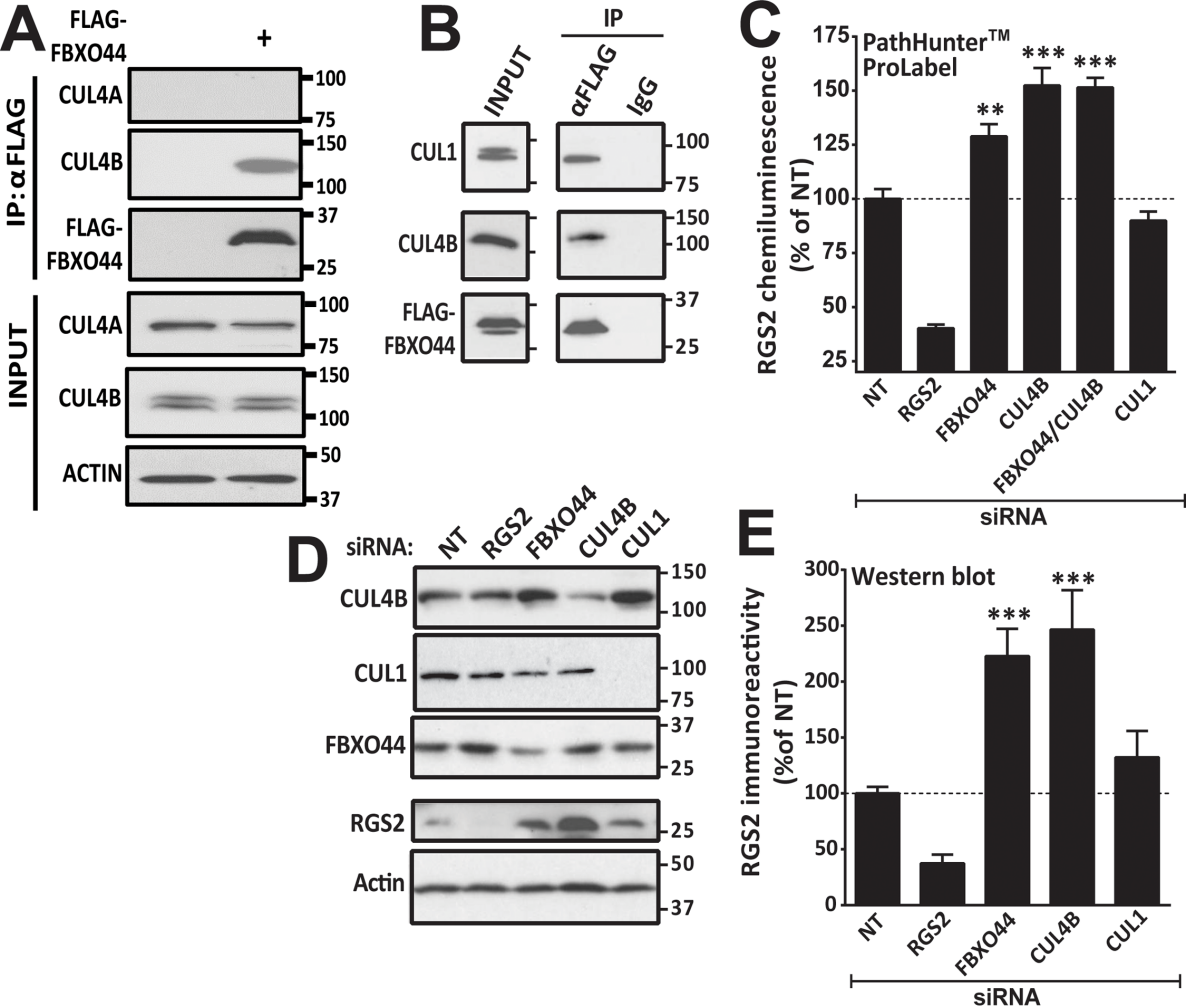
**A**. Endogenous CUL4B, but not CUL4A is co-immunoprecipitated with FLAG-FBXO44 in HEK-293T cells. **B**. FLAG-FBXO44 associates with both the canonical SCF component CUL1 as well as CUL4B. As shown with both the PathHunter ProLabel assay (**C**) and Western blot (**D, E**), CUL1 siRNA has no significant effect on RGS2 protein levels. There is no additive effect of simultaneous siRNA knockdown of FBXO44 and CUL4B. Data are presented as mean ± S.D. of 3 independent experiments run in triplicate; ***P<0.01; ***P<0.001 using one-way ANOVA with Bonferroni’s post hoc test for pairwise comparisons.

F-box proteins are generally associated with Skp1 and CUL1 in SCF (Skp-cullin-F-box) complexes and association of an F-box protein with CUL4 has not previously been described. Hence, we next investigated whether FBXO44 could associate with CUL4B. Indeed, FLAG-FBXO44 could co-immunoprecipitate CUL4B (Fig 4A). The closely related CUL4A did not co-immunoprecipitate with FLAG-FBXO44, despite ample protein expression of CUL4A.

As expected FLAG-FBXO44 also co-immunoprecitated its canonical interaction partner CUL1 (Fig 4B). We therefore used CUL1 siRNA to see if RGS2 could also be regulated by CUL1. In contrast to FBXO44 and CUL4B, siRNA-mediated knockdown of CUL1 had no effect on RGS2 protein levels as demonstrated by both the PathHunter ProLabel assay (Fig 4C) and western blot (Fig 4D and 4E). Furthermore, simultaneous knockdown of CUL4B and FBXO44 had no additive effects on RGS2 protein levels (Fig 4C) suggesting that the two proteins regulate RGS2 protein levels through a shared mechanism.

### DDB1, but not Skp1, is involved in RGS2 protein degradation

**Fig 5:**
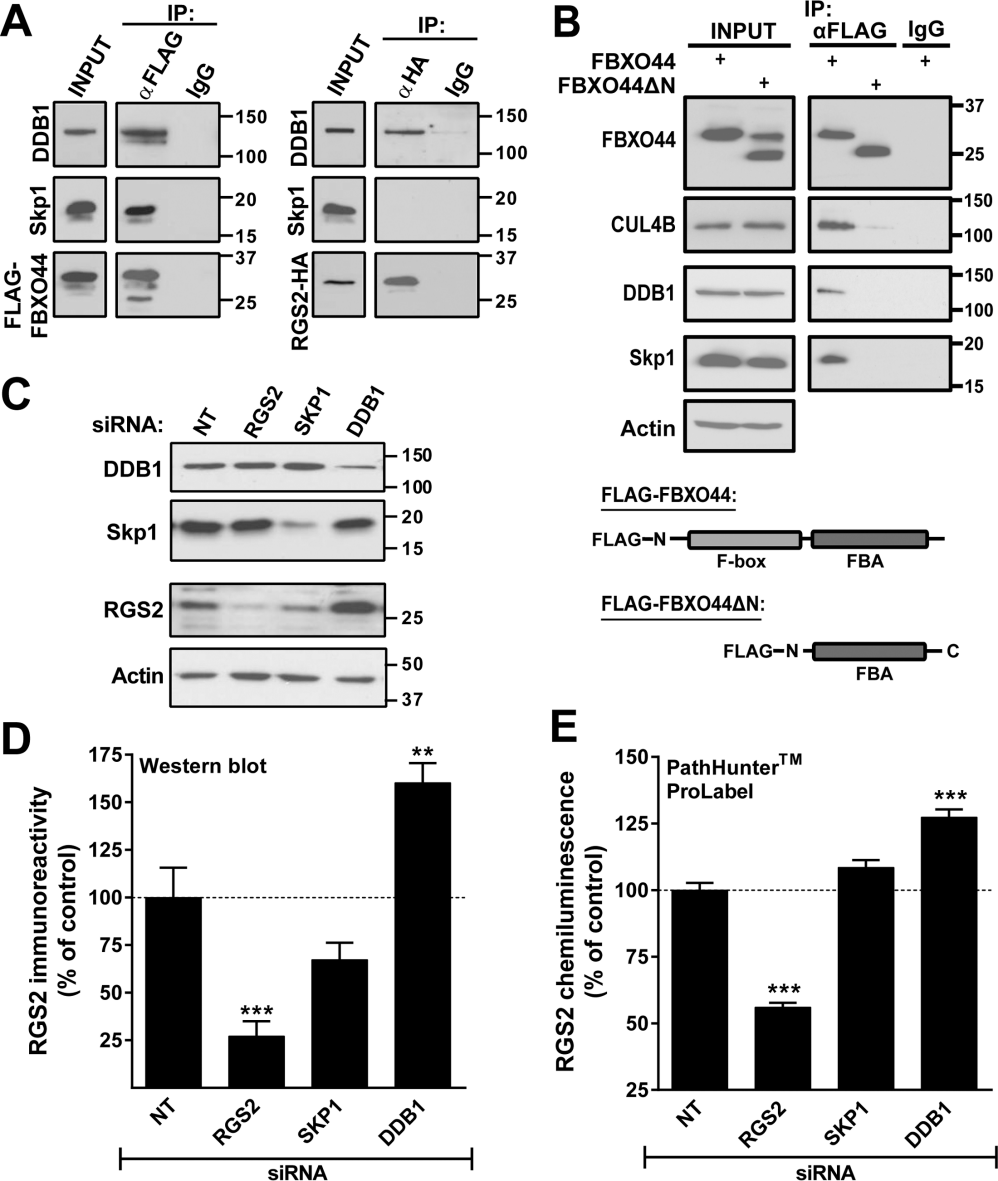
**A**. FLAG-FBXO44 associates with both Skp1 and DDB1 (left) whereas RGS2 only associates with DDB1 (right). **B**. The N-terminus and/or the F-box domain of FBXO44 is necessary for association with CUL4B, DDB1 and Skp1 as FBXO44ΔN does not co-immunoprecipitate either protein. Note that endogenous FBXO44 is recognized by the FBXO44 antibody used for Western blot. Hence, two bands (endogenous full length and transiently expressed FBXO44ΔN) are detected in the input. As shown with both Western blot (**C, D**) and the PathHunter ProLabel assay (**E**) DDB1 siRNA causes a significant increase in RGS2 protein levels whereas knockdown of Skp1 has no effect. Data are presented as mean ± S.D. of 3 independent experiments run in triplicate; **P<0.01; ***P<0.001 using one-way ANOVA with Bonferroni’s post hoc test for pairwise comparisons.

In the classical SCF complex, Skp1 serves as the link between CUL1 and F-box proteins. In CUL4B complexes, DNA damage binding protein 1 (DDB1) serves a similar role. Neither Skp1 nor DDB1 were among the genes included in the primary screen. We therefore investigated whether Skp1 or DDB1 would associate with RGS2 and regulate its protein degradation. Co-immunoprecipitation using FLAG-FBXO44 demonstrated that both Skp1 and DDB1 can associate with FBXO44 in cells (Fig 5A; left). However, RGS2-HA associated only with DDB1 (Fig 5A; right). We also looked at the role of the N-terminus and F-box domain of FBXO44 for association with Skp1, DDB1 and CUL4B. In contrast to the association with RGS2 (Fig 3E), the N-terminal region and/or the F-box domain of FBXO44 is essential for association with Skp1, DDB1 and CUL4B (Fig 5B) as only full length but not FBXO44ΔN was able to co-immunoprecipitate these three proteins. This is in line with previous literature demonstrating that the F-box domain is responsible for binding to Skp1 and suggests that FBXO44 also associates with CUL4B-DDB1 complexes through its N-terminal region.

We next investigated the effects of siRNA knockdown of Skp1 and DDB1 on RGS2 protein levels. siRNA-mediated knockdown of DDB1 significantly increased RGS2 protein levels, while reducing Skp1 expression had no effect on RGS2 protein levels (Fig 5C–5E).

**Fig 6:**
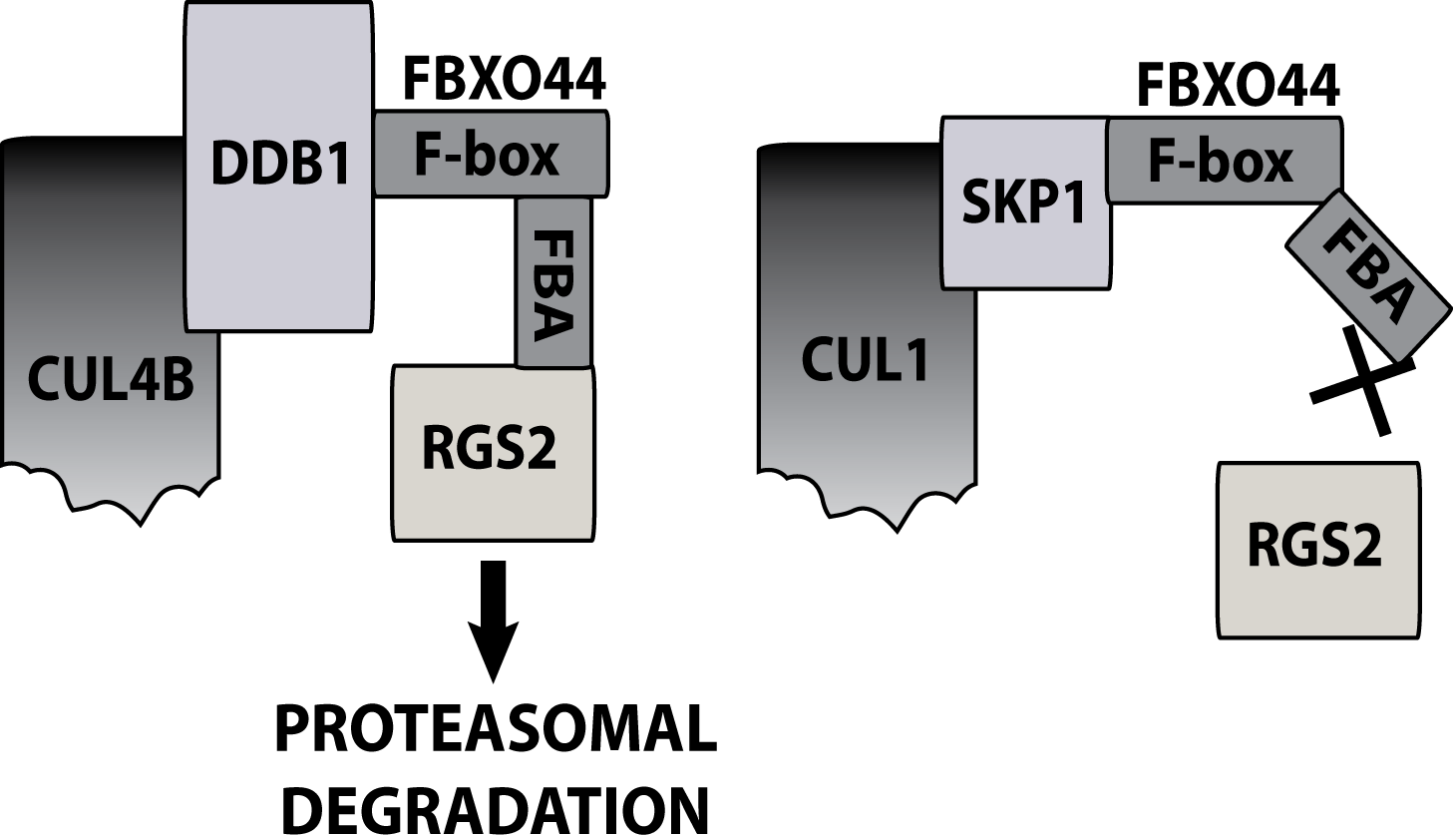
Model of a CUL4B/DDB1/FBXO44 E3 ligase complex responsible for RGS2 protein degradation. FBXO44 also associates with a CUL1/Skp1 complex, however this complex is unable to regulate RGS2 protein degradation.

Taken together our data provide strong evidence that RGS2 protein degradation is mediated by a novel CUL4B-DDB1-FBXO44 E3 ligase. Although FBXO44 also associates with a CUL1-Skp1 complex, this E3 ligase complex appears to be incapable of recruiting and degrading RGS2 (Fig 6).

## Discussion

Over the past decade it has become clear that low RGS2 protein levels are associated with diseases as diverse as hypertension [30,49], heart failure [50–52], prostate cancer [31], and anxiety [32,33]. Also, protein degradation can be a major mechanism to reduce RGS2 protein levels. Wild-type RGS2 has a very short protein half-life [35] and human SNPs associated with hypertension (e.g. Q2L in a Japanese population [53]) are characterized by low protein levels in vitro [36]. Pharmacologically increasing RGS2 protein levels has functional effects on GPCR signaling [35] suggesting that inhibiting RGS2 protein degradation could have broad therapeutic applications. The RGS2-FBXO44 protein-protein association identified here could be a novel site for small molecule modulators of RGS2 protein levels with much greater specificity than general proteasome inhibition.

Apart from RGS2, several other RGS proteins, including RGS4, 5 and 16 are substrates for rapid proteasomal degradation [36,37]. Indeed, our lab previously showed that both RGS2 and RGS4 are degraded through the proteasome [36] and that RGS4 seems to be more heavily regulated as demonstrated by lower basal protein levels and a larger effect of the proteasome inhibitor MG-132. In the present study we did not observe a difference in the magnitude of MG-132-induced increases in RGS2 vs. RGS4 (Fig 1A), however the basal protein levels of RGS4 was lower than RGS2 also in our study. The difference between the two studies can, however, be accounted for by the use of different treatment duration and concentration of MG-132. Bodenstein *et al*. conducted all experiments using 20 μM MG-132 for 4 hours [36] whereas in our current study we used 10 μM overnight (16–18 hours), when a single concentration was chosen.

RGS4 and 5 are well-known substrates for the N-end rule pathway and the Harper lab demonstrated that RGS4 is degraded by the N-degron-specific E2/E3 pairs USE1/UBR1 and UBE2/UBR1 [39]. We now show that RGS2 is ubiquitinated (Fig 1C) and subject to rapid degradation by the proteasome [35,36]. Despite the similarities, RGS2 and RGS4 clearly utilize distinct pathways for proteasomal targeting. Our results define a novel CRL complex involved in RGS2 degradation.

To date over 600 E3 ligases have been identified and they are divided into several classes of which the CRL family is by far the largest (reviewed in [54–56]). The current study identifies a novel substrate for CUL4B, RGS2, which is not regulated by the very closely related CUL4A. Despite the high similarity between these proteins (80% sequence homology), several other CUL4B-specific substrates have been identified previously, including Peroxiredoxin III [46], Jab5 [57] and Histone H2A [45]. In contrast, substrates specific for CUL4A have yet to be identified.

F-box proteins are the substrate-recognizing component of SCF complexes [47] and to date over 50 mammalian F-box proteins have been identified. FBXO44 is a member of the FBA family (FBXO2, FBXO6, FBXO17, FBXO27 and FBXO44) [58] which are all predicted to bind glycoproteins through the C-terminal domain. However, despite the high sequence similarity with the other members, FBXO44 does not bind these substrates [58]. Recently BRCA1 was identified as a novel substrate for FBXO44 [59]. Low protein expression of BRCA1 is known to promote breast- and ovarian cancers and FBXO44 promotes ubiquitination and degradation of BRCA1. Furthermore, high levels of FBXO44 (and low levels of BRCA1) could be found in human breast cancer tumors [59] providing a rationale for targeting FBXO44. Interestingly, Lyu *et al*. found RGS2 mRNA levels to be down-regulated in both human breast cancer tissue as well as in invasive breast cancer cell lines [42]. Furthermore, overexpression of RGS2 could suppress cancer cell growth. They also identified that the deubiquitinating enzyme (DUB) MCPIP1 can stabilize RGS2 protein. This finding, together with our present results, suggests that RGS2 protein stability could be a promising avenue in the treatment of breast cancer. It also sheds light on how RGS2 protein levels are regulated by the ubiquitin-proteasome system.

While FBXO44 does interact with its canonical complex CUL1/Skp1, it also associates with CUL4B/DDB1. Surprisingly, only the latter complex is capable of recruiting and degrading RGS2 protein. The mechanism for this specificity is unclear. Binding of FBXO44 to Skp1 or DDB1, respectively could cause distinct conformations to either favor or inhibit RGS2 protein binding (see Fig 6). Indeed, Skp1 has previously been suggested to stabilize conformations of F-box proteins that favor substrate binding [60] and it is feasible to suggest that the same is true for DDB1.

Further biochemical studies are needed to characterize the association between FBXO44 and CUL4B-DDB1 on a molecular level. CUL4-DDB1 complexes can interact with proteins containing an F-box domain but generally in the context of a WD40 motif [61]. Hu et al. showed that FBW5 (containing both WD40 and F-box domains) can promote degradation of the tumor suppressor tuberous sclerosis gene 2 through association with a CRL4 complex [62]. Our study is the first to identify a CUL4-F-box protein association in the absence of a WD40 domain, further adding complexity to the CRL E3 ligase system. The canonical Gα-RGS2 interaction raises questions in this regard. Gβ contains WD40 motifs that are crucial to their function [63]. Based on our experimental results we cannot exclude the possibility that other proteins are mediating the interaction between FBXO44 and RGS2. Further studies are needed to elucidate this mechanism.

RGS2 is highly expressed in brain, heart, kidney and vascular smooth muscle cells. A genome-wide screen of mRNA transcripts previously revealed that FBXO44, CUL4B and DDB1 seem to be widely expressed, including in tissues where RGS2 is also present [64]. In the current study we demonstrated that RGS2 associates with FBXO44 in heart tissue. Previous literature indicates that FBXO44 is also expressed in the brain but not the kidney [58], opening up the possibility for tissue-specific regulation of RGS2 protein levels. However, the previous study also did not detect FBXO44 protein in the heart. Hence, the tissue distribution of FBXO44 still needs complete elucidation.

In conclusion, we have defined a novel multiprotein E3 ligase capable of RGS2 degradation, where F-box substrate specificity is uniquely dependent on a CUL4B/DDB1 context. Future studies will further dissect the molecular basis of RGS2 protein degradation and evaluate this mechanism as a potential druggable target.

## Supporting Information

S1 Table Bioinformatic classification of hits from siRNA screen for genes that regulate RGS2 protein expression. (DOCX)

S2 Table Genes involved in ubiquitin-proteasome-mediated protein degradation that were primary hits in the siRNA screen for up-regulation of RGS2 protein expression. (DOCX)
