# Supplementary material for: FBXO44-Mediated Degradation of RGS2 Protein Uniquely Depends on a Cullin 4B/DDB1 Complex
Source: PLoS One. 2015 May 13;10(5):e0123581. doi: 10.1371/journal.pone.0123581 (PMC4430315; doi:10.1371/journal.pone.0123581)
Supplement: S1 Table — (DOCX) [file pone.0123581.s001.docx]

**TABLE S1. Bioinformatic classification of hits from siRNA screen for genes that regulate RGS2 protein expression.** Hits in the primary siRNA screen were subjected to analysis and classification using ConceptGen as described in Supplemental methods. The 14 most enriched classes of biological/molecular function are displayed. Nine of the 14 are related to either protein kinase activity or proteolysis.

| Concept Name | Genes in screen | Hits in screen | Hit rate (%) /concept | P-Value |
| --- | --- | --- | --- | --- |
| Kinase activity | 790 | 30 | 3.8 | 1.96E-12 |
| Phosphate metabolic process | 940 | 32 | 3.4 | 1.71E-11 |
| Peptidase activity | 570 | 22 | 3.9 | 3.81E-09 |
| Protein amino acid phosphorylation | 653 | 24 | 3.7 | 3.66E-09 |
| Proteolysis | 676 | 23 | 3.4 | 3.58E-08 |
| Ubiquitin cycle | 473 | 17 | 3.6 | 2.05E-06 |
| Modification-dependent protein catabolic process | 176 | 10 | 5.7 | 1.71E-05 |
| Proteolysis involved in cellular protein catabolic process | 177 | 10 | 5.6 | 1.79E-05 |
| Cellular protein catabolic process | 179 | 10 | 5.6 | 1.95E-05 |
| Insulin signaling pathway | 138 | 9 | 6.5 | 2.57E-05 |
| Ion channel activity | 365 | 12 | 3.3 | 1.69E-04 |
| Endopeptidase activity | 381 | 12 | 3.1 | 2.45E-04 |
| Passive transmembrane transporter activity | 383 | 12 | 3.1 | 2.57E-04 |
| G-protein coupled receptor activity | 863 | 18 | 2.1 | 5.08E-04 |
