# Supplementary material for: FBXO44-Mediated Degradation of RGS2 Protein Uniquely Depends on a Cullin 4B/DDB1 Complex
Source: PLoS One. 2015 May 13;10(5):e0123581. doi: 10.1371/journal.pone.0123581 (PMC4430315; doi:10.1371/journal.pone.0123581)
Supplement: S2 Table — (DOCX) [file pone.0123581.s002.docx]

**TABLE S2.** **Genes involved in ubiquitin-proteasome-mediated protein degradation that were primary hits in the siRNA screen for up-regulation of RGS2 protein expression.** Genes are ranked according to degree of RGS2 protein increase when knocked down. Confirmation was performed with the single siRNA oligos from each siGENOME SMART-POOL. At least two of the four oligos needed to cause a significant increase in RGS2 protein levels to confirm.

| Gene ID | Gene Name | Gene Symbol | Confirmed |
| --- | --- | --- | --- |
| 8450 | Cullin 4B | *CUL4B* | **++** |
| 93611 | F-box protein 44 | *FBXO44* | **++** |
| 494188 | F-box protein 47 | *FBXO47* | **-** |
| 84708 | Ligand of numb-protein X 1 | *LNX1* | **-** |
| 9867 | Praja ring finger 2 | *PJA2* | **-** |
| 5690 | Proteasome (prosome, macropain) subunit, beta type, 2 | *PSMB2* | **-** |
| 5695 | Proteasome (prosome, macropain) subunit, beta type, 7 | *PSMB7* | **-** |
| 115201 | Autophagy related 4 homolog A (S. cerevisiae) | *ATG4* | **+** |
| 580 | BRCA1 associated RING domain 1 | *BARD1* | **-** |
| 283450 | chromosome 12 open reading frame 51 | *C12orf51* | **+** |
| 5527 | protein phosphatase 2, regulatory subunit B', gamma isoform | *PPP2R5C* | **-** |
| 26994 | Ring finger protein 11 | *RNF11* | **-** |
| 165918 | Ring finger protein 168 | *RNF168* | **+** |
| 10273 | STIP1 homology and U-box containing protein 1 | *STUB1* | **-** |
| 127247 | Ankyrin repeat and SOCS box-containing 17 | *ASB17* | **+** |
| 7353 | Ubiquitin fusion degradation 1 like (yeast) | *UFD1L* | **-** |
| 57478 | Ubiquitin specific peptidase 31 | *USP31* | **-** |
| 124739 | Ubiquitin specific peptidase 43 | *USP43* | **+** |
| 7336 | Ubiquitin-conjugating enzyme E2 variant 2 | *UBE2V2* | **-** |
| 3093 | Ubiquitin-conjugating enzyme E2K (UBC1 homolog, yeast) | *UBE2K* | **-** |
| 55884 | WD repeat and SOCS box-containing 2 | *WSB2* | **+** |

++ >3 S.D vs. non-targeting siRNA; + >2 S.D vs. non-targeting siRNA
